# “Monsieur Tonsin Come Again”—Rubber Subject

**Published:** 1872-02

**Authors:** 


### "MONSIEUR TONSIN COME AGAIN"—RUBBER SUBJECT.

The expiration of the Goodyear patent for the vulcanization of rubber occurs in May next, when we suppose anybody can vulcanize rubber or any allied gum, for any or all purposes, without let or hindrance, except the thing formed should assume the shape of a plate for an artificial denture. Upon this particular thing a restriction is imposed—and why? Simply because, after the material and process had been demonstrated to be available as a base for artificial teeth, and used to some extent by the dental profession, it occurred to a certain individual to procure a patent for the application of vulcanized rubber to this purpose. Why the patent was granted we have ever been at a loss to know. A hundred other patents might have been granted upon the same grounds. Indeed, we think there would be quite as much justice in now granting a patent for the application of vulcanized rubber in the manufacture of baby whistles; but we are not now writing to give our views upon the rubber question, or the Cummings patent, but as it is a matter of most vital importance to many in our profession, we simply aim to give, for the gratification of those interested, the little information we possess in reference to the present status of the question.

And in the first place, from intelligence gathered from various sources, it seems that the Dental Vulcanite Company have left no means unemployed to secure the validity of the Cummings patent, and the result is, they have considerably the start of the dental profession in the race. They have obtained some of the strongest counsel in the country. Colonel Fisher, the former advocate for the profession, than whom there is no stronger and more influential patent lawyer in the country, has been laid upon the shelf; others have been disposed of in the same way. This will be a matter of exceeding regret to the profession. Some time ago prosecution was instituted under the Cummings patent against Dr. B. E. Gardiner, of Providence, R. I., the result of which was a decision of the U. S. District Court affirming the validity of the Cummings patent. An appeal was taken in this case to the Supreme Court of the U. S., for the purpose of reversing the decision. It was set for a hearing in January, 1872. We have not learned that the case has yet been argued.

The dentists of New York and Brooklyn, and perhaps other places, have united in sustaining this appeal. Able counsel have been employed in New York and Philadelphia to conduct and argue the case before the Supreme Court. What the result of that appeal will be, time only will determine. If a decision sustaining the patent is obtained in this Court, that will end the contest in this direction.

There is a way, however, in which this can be very decisively met, viz.: STOP THE USE OF RUBBER, as every man in the profession ought to have done long ago, and as we think every one would if they had taken a correct view of the degraded position to which they were reduced, by being placed under the imperious and demoralizing exactions of the Dental Vulcanite Company.

That company has held a rod—yes, a "cat-o'-nine-tails"—over the heads of the yielding, timid, frightened members of the dental profession, until they have opened their purses and said take it.

Now, it is high time this procedure should stop, and the profession can stop it if they will, and the indications now are that they WILL. Meetings are being called at various places throughout the country to consider what is best to be done. A meeting of the profession of New York State is to be held in New York on the 13th inst., and of Pennsylvania in Philadelphia on the 27th, and of the Western States in Cincinnati on the 7th of March prox., as will be seen by circular in advertising sheet of this month. It is desirable that these meetings should be largely attended, and let the action be prompt and decisive, and such as can not be mistaken.

The question comes every day, shall I take out license for this year, and under the Cummings patent? Let the answer ring everywhere, NO.

